# Five Dissertations on Fever

**Published:** 1846-06

**Authors:** 


Art. VIII.—*Five Dissertations on Fever.* By George Fordyce, M. D., F.R.S., Fellow of the Royal College of Physicians; Senior Physician to St. Thomas's Hospital, and Reader on the Practice of Physic in London. Second American edition with an Introduction. Philadelphia: Ed. Barrington & Geo. D. Haswell. 1846. 8vo. pp. 403

This celebrated work, the author of which was born nearly a hundred and ten years ago, appears for the second time before the profession of this country, having been issued as a number of the *Select Medical Library.* The Introduction consists of an interesting biographical sketch of the author by Dr. John Bell, editor of the Library. A very bad style, tedious, at the same time that it is wanting in smoothness, defective punctuation, and an arrangement which rendered it troublesome to refer to any particular passage in the Dissertations, account for their want of popularity. That they possess much intrinsic value, and are entitled to maintain a place among the many treatises which fever has since called forth, is the judgment of the best informed medical readers and critics. In a word, the work is entitled to take its rank among the classics in medicine, and we are ready to acknowledge the debt under which Dr. Bell has brought us by its republication; which will carry many a reader back with us to the days of our pupilage, when "Fordyce on Fever" made a part of the compact little library from which we were wont to derive instruction. Besides adding the Introduction, the American Editor has sub-divided the Dissertations, and affixed to each division a full table of contents, by means of which the difficulty of consulting the work is obviated. The number of practitioners is not small who will be glad of an opportunity of adding this volume to their stock of medical books.

